# BNT162b2 COVID-19 vaccination in children alters cytokine responses to heterologous pathogens and Toll-like receptor agonists

**DOI:** 10.3389/fimmu.2023.1242380

**Published:** 2023-08-25

**Authors:** Andrés Noé, Thanh D. Dang, Christine Axelrad, Emma Burrell, Susie Germano, Sonja Elia, David Burgner, Kirsten P. Perrett, Nigel Curtis, Nicole L. Messina

**Affiliations:** ^1^ Infection, Immunity and Global Health, Murdoch Children’s Research Institute, Parkville, VIC, Australia; ^2^ Infectious Diseases Unit, The Royal Children’s Hospital, Melbourne, Parkville, VIC, Australia; ^3^ Department of Paediatrics, The University of Melbourne, Parkville, VIC, Australia; ^4^ Population Health, Murdoch Children’s Research Institute, Parkville, VIC, Australia

**Keywords:** SARS-CoV-2, heterologous, immunisation, paediatric vaccination, innate immunity, off-target effects, BNT162b2, mRNA vaccination

## Abstract

**Background:**

Vaccines can have beneficial off-target (heterologous) effects that alter immune responses to, and protect against, unrelated infections. The heterologous effects of COVID-19 vaccines have not been investigated in children.

**Aim:**

To investigate heterologous and specific immunological effects of BNT162b2 COVID-19 vaccination in children.

**Methods:**

A whole blood stimulation assay was used to investigate *in vitro* cytokine responses to heterologous stimulants (killed pathogens, Toll-like receptor ligands) and SARS-CoV-2 antigens. Samples from 29 children, aged 5-11 years, before and 28 days after a second BNT162b2 vaccination were analysed (V2 + 28). Samples from eight children were analysed six months after BNT162b2 vaccination.

**Results:**

At V2 + 28, interferon-γ and monocyte chemoattractant protein-1 responses to *S. aureus*, *E. coli*, *L*. *monocytogenes*, BCG vaccine, *H. influenzae*, hepatitis B antigen, poly(I:C) and R848 stimulations were decreased compared to pre-vaccination. For most of these heterologous stimulants, IL-6, IL-15 and IL-17 responses were also decreased. There were sustained decreases in cytokine responses to viral, but not bacterial, stimulants six months after BNT162b2 vaccination. Cytokine responses to irradiated SARS-CoV-2, and spike glycoprotein subunits (S1 and S2) were increased at V2 + 28 for most cytokines and remained higher than pre-vaccination responses 6 months after BNT162b2 vaccination for irradiated SARS-CoV-2 and S1. There was no correlation between BNT162b2 vaccination-induced anti-SARS-CoV2-receptor binding domain IgG antibody titre at V2 + 28 and cytokine responses.

**Conclusions:**

BNT162b2 vaccination in children alters cytokine responses to heterologous stimulants, particularly one month after vaccination. This study is the first to report the immunological heterologous effects of COVID-19 vaccination in children.

## Background

In addition to antigen-specific adaptive immunity to the target pathogen and cross-protective immunity to related microbes (e.g., protection against *Mycobacterium tuberculosis* and *Mycobacterium leprae* induced by *Mycobacterium bovis*-derived bacille Calmette–Guérin (BCG)) (1), vaccines have off-target (heterologous) effects that protect against unrelated pathogens (2–4).

In high-mortality settings, live-attenuated vaccines are associated with reductions in all-cause infant mortality greater than can be attributed to vaccine-specific protection alone (5–7). The reduction in all-cause mortality in high-mortality settings is proposed to be due, at least in part, to protection against infections unrelated to the vaccine target (2–4). Trained immunity, the process by which innate immune cells such as monocytes develop immunological memory through metabolic and epigenetic changes, is one proposed mechanism by which vaccines exert heterologous effects (8, 9). Understanding heterologous effects and trained immunity, and harnessing positive heterologous effects has the potential to extend vaccine-induced protection to a diverse array of pathogens.

The COVID-19 pandemic has prompted a resurgence of interest in the heterologous effects of BCG and other vaccines and compounds (10–14). Heterologous immunological effects following vaccination have been explored in several studies by assessing *in vitro* cytokine responses to heterologous antigens (9, 15–19). Two small studies have reported on heterologous effects of COVID-19 vaccines. One study in adults reported that following adenoviral COVID-19 (ChAdOx1) vaccination, monocyte proinflammatory cytokine and chemokine production and glycolysis is enhanced in resting states as well as in response to unrelated stimulants (20). COVID-19 mRNA-based vaccines have been reported to modulate transcriptional profiles in monocytes from adults (21). To date, the heterologous effects of COVID-19 vaccines have not been investigated in children.

In the COVID-19-Specific vaccine and heterologous Immunity in MIS BAIR (COSI BAIR) study (22), we investigated the heterologous and specific immunological effects of BNT162b2 COVID-19 vaccination in children.

## Methods

### Study design, participants, and recruitment

Participants of COSI BAIR were a subset of children from the Melbourne Infant Study: BCG for Allergy and Infection Reduction (MIS BAIR) randomised-controlled trial (ClinicalTrials.gov identifier NCT01906853) (22). MIS BAIR investigated whether neonatal BCG vaccination protected against infant and childhood infection, allergy, and asthma. The inclusion and exclusion criteria for MIS BAIR are described elsewhere (22). COSI BAIR was approved by the research ethics committees of the Royal Children’s Hospital, Melbourne (HREC/81771/RCHM-2021). COSI BAIR was registered at ClinicalTrials.gov (NCT05168709) and was monitored by an internal monitor and an independent safety monitor. A parent and/or legal guardian of each participant gave informed consent during the enrolment process.

**Figure 1:**
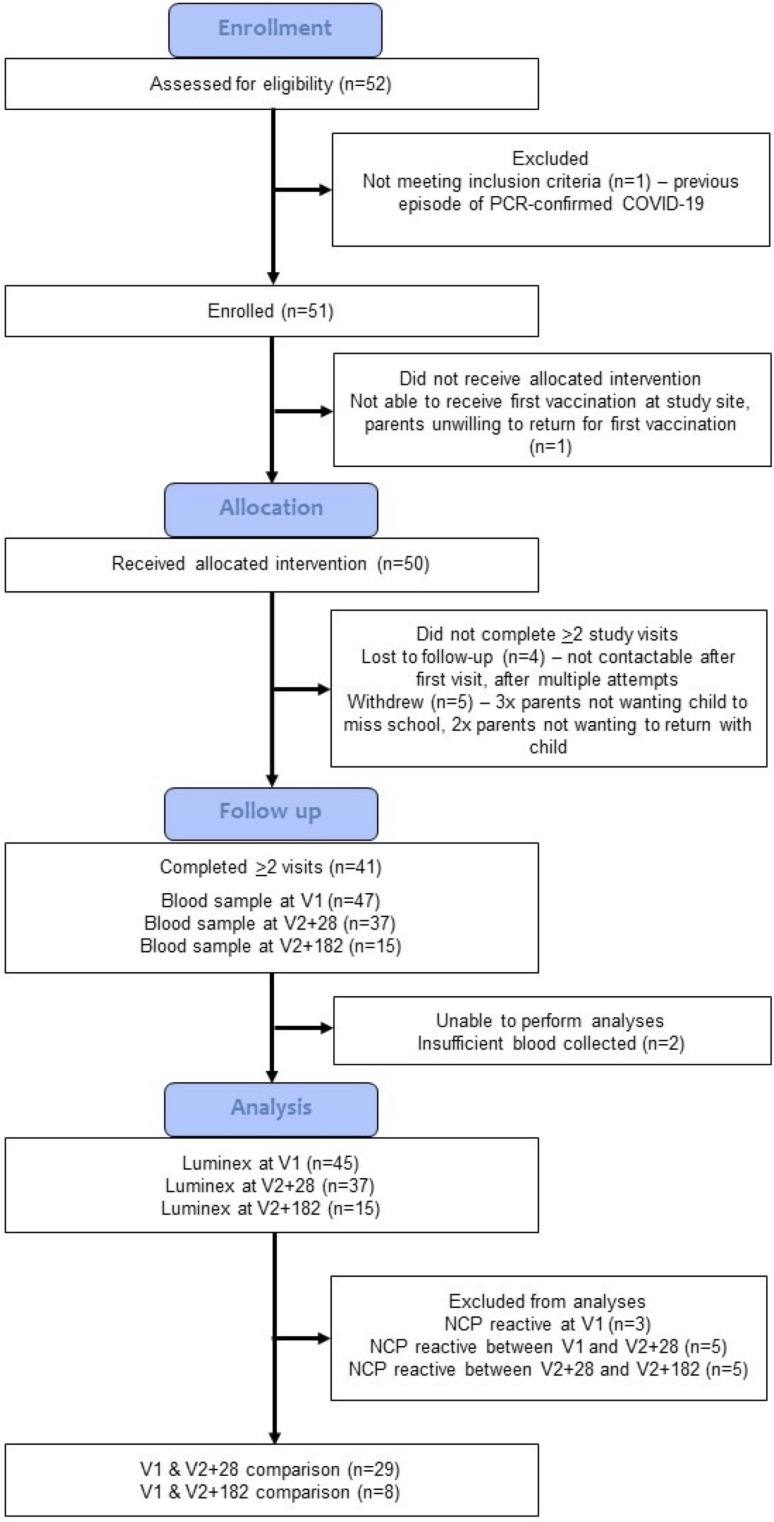
Study CONSORT diagram. The COVID-19-Specific vaccine and heterologous Immunity in MIS BAIR (COSI BAIR) study population is a selection from the BCG for Allergy and Infection Reduction (MIS BAIR) clinical trial. A total of 51 participants were enrolled into COSI BAIR.

As part of MIS BAIR, neonates were randomised in the first 10 days of life to receive BCG-Denmark vaccination or no BCG vaccination; participants from both groups were recruited into COSI BAIR (**Figure 1**). Inclusion criteria for COSI BAIR included age between five and eleven years and consent in MIS BAIR to be contacted about future research. Exclusion criteria included hypersensitivity to BNT162b2, previous receipt of a COVID-19 vaccine, previous polymerase chain reaction test-confirmed COVID-19, clinically significant medical morbidity, a parent and/or legal guardian unwilling to give consent for blood samples to be taken from their child at each study visit and receipt of BCG vaccine at any other time than as part of the MIS BAIR trial. These exclusion criteria were selected to ensure the safety of trial participants and maximise the internal validity of the study.

### Intervention

COSI BAIR was a single arm clinical trial in which all participants received the COMIRNATY COVID-19 vaccine (BNT162b2). Participants received two doses, 8 weeks apart, of 10 μg (in 0.2 mL) BNT162b2 (Pfizer, NY, USA). The vaccine, dose, interval, and schedule were approved by the Therapeutic Goods Administration of Australia and recommended by the Australian Technical Advisory Group on Immunisation (ATAGI), and therefore were the standard of care. All first vaccinations were done by the Royal Children’s Hospital, Melbourne Immunisation Service. For 8 participants, the parent and/or legal guardian elected for their child to receive the second dose of the vaccine at their local medical service provider as part of the routine vaccination programme. These participants remained enrolled in the COSI BAIR trial and were otherwise followed up as per the protocol. All participants received two doses of BNT162b2 vaccine in accordance with ATAGI guidance.

### Sample collection

Participants were requested to provide blood samples at two core visits, and one optional visit. The first blood sample was taken immediately before, and on the same day as, the first BNT162b2 vaccination (V1), the second blood sample was taken 28 days after the second BNT162b2 vaccination (V2 + 28) and the optional third blood sample was taken 6 months after the second BNT162b2 vaccination (V2 + 182) (**Supplementary Figure S1**). Up to 23 mL venous blood was collected into sodium heparin-containing and serum separator tubes (Becton Dickinson, NJ, USA).

### *In vitro* whole blood stimulation

*In vitro* whole blood stimulation assays were done as previously described (16, 23). Briefly, within 2 hours of blood collection, whole blood diluted 1:1 with RPMI 1640 medium (GlutaMAX Supplement, HEPES, Gibco, Life Technologies), was stimulated at 37°C with 5% CO_2_ for 20 (± 2) hours with pre-prepared stimulants. Following stimulation, the supernatants were harvested and stored at -80°C prior to analysis. The stimulant conditions included an unstimulated control (RPMI medium alone) and SARS-CoV-2 stimulants: irradiated un-infected-Vero cell supernatants (iVERO) 1:10, irradiated SARS-CoV-2 infected-Vero cell supernatants 10^6.2^ TCID_50_/mL (iSARS; hCoV‐19/Australia/VIC01/2020 (VIC01, NCBI: MT007544.1, virus was a clinical isolate kindly gifted by the Victorian Infectious Diseases Reference Laboratory (VIDRL) and iVERO and iSARS kindly gifted by Prof Kanta Subbarao and Dr Rajeev Rudraraju of the The Peter Doherty Institute for Infection and Immunity and WHO Collaborating Centre for Reference and Research on Influenza), SARS-CoV-2 spike glycoprotein S1 subunit antigen (S1; Abcam, UK) 5 μg/mL, SARS-CoV-2 spike glycoprotein S2 subunit antigen (S2; Abcam, UK) 5 μg/mL and SARS-CoV-2 nucleocapsid protein (NCP) antigen (Abcam, UK) 5 μg/mL. Other stimulants have previously been described (16) and included: bacterial stimulants (heat-killed (HK) *Haemophilus influenzae* type B 1.0x10^6^ CFU/mL, HK *Listeria monocytogenes* 1.0x10^7^ CFU/mL, BCG-Denmark (Serum Statens Institut, Denmark) 75 µg/mL, HK *Staphylococcus aureus* 1.0x10^7^ CFU/mL and HK *Escherichia coli* 1.0x10^6^ CFU/mL), and viral/other stimulants (hepatitis B virus surface antigen 3.5 μg/mL; (GSK, Australia), resiquimod (R848; TLR7/8 agonist; Sigma-Aldrich) 3.5 μg/mL, HK *Candida albicans* 1.0x10^6^ CFU/mL, and polyinosinic-polycytidylic acid (poly(I:C); TLR3 agonist) 5 μg/mL. If insufficient blood was available for all stimulation conditions, a predetermined priority order was used.

Supernatants were analysed in batches using Bio-Plex Pro Human Cytokine 27-plex Assay (Bio-Rad, California, USA) kits following the manufacturer’s instructions. The assay allowed detection of 27 cytokines and chemokines in each well: Eotaxin, Basic fibroblast growth factor basic (FGF), granulocyte-colony stimulation factor (G‐CSF), granulocyte macrophage colony-stimulating factor (GM‐CSF), interferon-γ (IFN‐γ), Interleukin (IL)‐1β, IL‐1rα, IL‐2, IL‐4, IL‐5, IL‐6, IL‐7, IL‐8, IL‐9, IL‐10, IL‐12(p70), IL‐13, IL‐15, IL‐17, IFN-γ-induced protein (IP‐10), monocyte chemoattractant protein (MCP‐1), macrophage inflammatory protein (MIP)‐1α, MIP‐1β, platelet-derived growth factor-BB (PDGF‐BB), regulated on activation, normal T cell expressed and secreted (RANTES), tumour necrosis factor-α (TNF‐α), and vascular endothelial growth factor (VEGF). This assay was selected to provide a broad screening panel consisting of biologically relevant adaptive immunity, pro-inflammatory and anti-inflammatory cytokines important to vaccine-induced and infectious disease immunity. The mean fluorescence intensity was read for each cytokine using a Bio-Plex 200 System using the Bio‐Plex Manager™ 6.1 software (Bio‐Rad). Each sample was analysed at a 1:10 dilution to account for the anticipated dynamic range of the cytokine results, as indicated by optimisation experiments (data not shown).

### SARS-CoV-2 serology

Plasma was isolated from whole blood collected in sodium heparin tubes (Becton Dickinson, NJ, USA) and stored at −80°C until analysis. SARS-CoV-2 NCP antibody testing of plasma samples was done by VIDRL using a commercially available anti-SARS-CoV-2 NCP enzyme-linked immunosorbent assay (ELISA) (Euroimmun Medizinische Labordiagnostika, Lübeck, Germany). This assay detects IgG antibodies against SARS-CoV-2 NCP protein and was done according to the manufacturer’s recommendations.

Serum was isolated from whole blood collected in serum separator tubes (Becton Dickinson, NJ, USA) and stored at −80°C until analysis. SARS-CoV-2 spike protein and receptor-binding domain (RBD) were quantified by ELISA at University of Adelaide and Basil Hetzel Institute for Translational Health Research as previously described (24, 25).

### Statistical analysis

The primary outcome of this study was the difference in *in vitro* whole blood stimulation cytokine responses to SARS-CoV-2-heterologous and specific stimulants between V1 and V2 + 28. Those with previous SARS-CoV-2 infection (identified by serum antibody reactivity against the NCP antigen) were excluded from final analyses (**Figure 1**). As it had been more than 5 years since children received or did not receive neonatal BCG vaccination as part of the MIS BAIR trial, BCG vaccination status was not included in the analyses. A sensitivity analysis of the primary outcome was done using data only from participants who did not receive BCG vaccination.

Cytokine results below the lower limit of detection were assigned a value of half the lowest detectable value (either the lowest standard on the curve or the lowest extrapolated value below). There was a total of 232 data points above the limit of detection out of 30,294 points from the 27 cytokines and 15 stimulation conditions measured (**Supplementary Table S1**); these were excluded from the analysis. There was a high degree of standardisation in laboratory procedures (**Supplementary Figure S1**, **Supplementary Table S2**). Prior to analysis, cytokine data were log-transformed to attain normal distributions. Cytokine concentration in response to each stimulant was background-corrected (iVERO condition was subtracted from iSARS stimulations; media only condition for other stimulants). Differences between log-transformed cytokine values at V1 and V2 + 28 were assessed for statistical significance using paired t-tests. Differences between log-transformed cytokine values at V1 and V2 + 182, and V2 + 28 and V2 + 182 were assessed for statistical significance using the Wilcoxon signed-rank test. There was interindividual variability in cytokine responses under stimulated and unstimulated conditions, typical of cytokine data (**Supplementary Figures S2A-E**). Spearman’s rank correlation coefficient was used to assess the significance of correlations between the V1 to V2 + 28 difference in anti-RBD IgG antibody titre and the V1 to V2 + 28 difference in the logarithm (log(V2 + 28) - log(V1)) of cytokine responses.

Statistical analysis was done using Stata version 17.0 (StataCorp LP, College Station) and visualisations generated using GraphPad Prism version 9.0 (GraphPad Software, San Diego).

## Results

### Study population

A total of 112 children were pre-screened for eligibility, of whom 37 had already received a COVID-19 vaccine and 18 did not reply to further contact. Of 52 children invited by the trial team to be assessed for eligibility at the first visit, 51 were eligible for inclusion and enrolled in the trial (**Figure 1**). Enrolment was completed between 20 January and 1 February 2022. Fifty children received the BNT162b2 vaccine as part of the COSI BAIR trial. Overall, the median age at first BNT162b2 vaccination was 6.4 years (IQR = 5.88-6.83) (**Supplementary Table S2**). Of the 47 children who provided a blood sample at V1, 21 were female and 32 did not receive BCG in the MIS BAIR trial. Three children were SARS-CoV-2 NCP reactive at the first visit, eight at V2 + 28 and seven at V2 + 182, all were excluded in the primary analysis. Therefore, 29 children with paired samples were included in the final analyses.

### BNT162b2 vaccination is associated with a decrease in bacterial and viral stimulant-induced cytokine responses one month after vaccination

**Figure 2:**
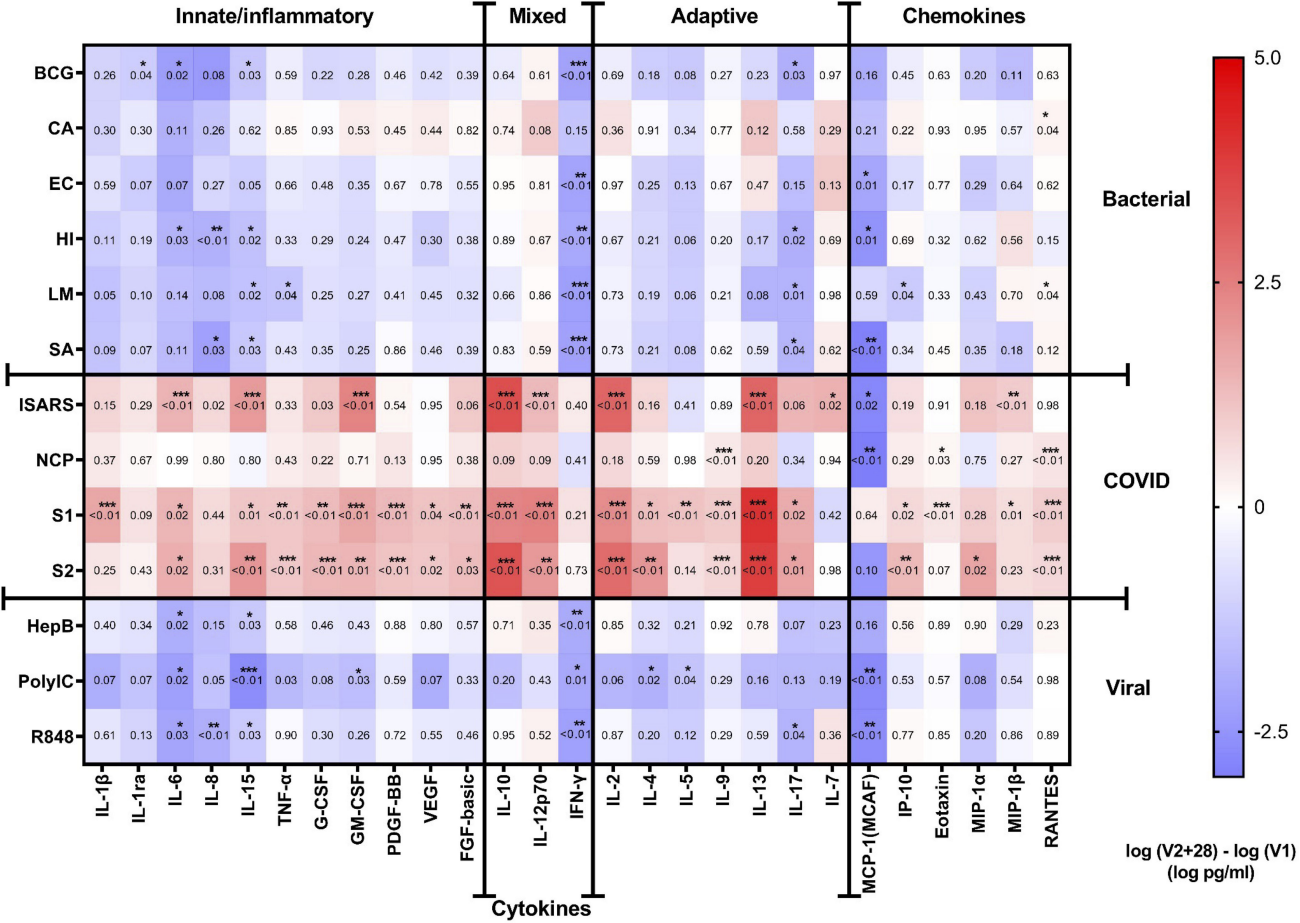
Cytokine responses to whole‐blood stimulations 28 days after BNT162b2 vaccination. The mean of the pre (V1) and post (V2 + 28) cytokine differences from 29 study participants is plotted in the heatmap colour gradient, with red representing increases and blue indicating decreases. Each row represents a stimulation condition, and is separated into three groups: bacterial/fungal stimulations (bacille Calmette-Guérin vaccine (BCG), *C. albicans* (CA), *E. coli* (EC), *H. influenzae* (HI), *L. monocytogenes* (LM), and *S. aureus* (SA)); SARS-CoV-2 stimulations (irradiated-SARS-CoV-2 (iSARS), SARS-CoV-2 nucleocapsid protein (NCP), SARS-CoV-2 spike Protein S1 (S1) and S2 (S2); and viral/TLR stimulations (hepatitis B antigen (HepB), polycytidylic acid (Poly(I:C)), resiquimod (R848)). Differences were assessed for statistical significance by paired t-tests of the difference in the logarithm of V1 and logarithm of V2 + 28 cytokine concentrations, p-values for each test are represented in respective boxes. Asterisks in the boxes depict significance (*p<0.05, **p<0.01 and ***p<0.001).

**Figure 3:**
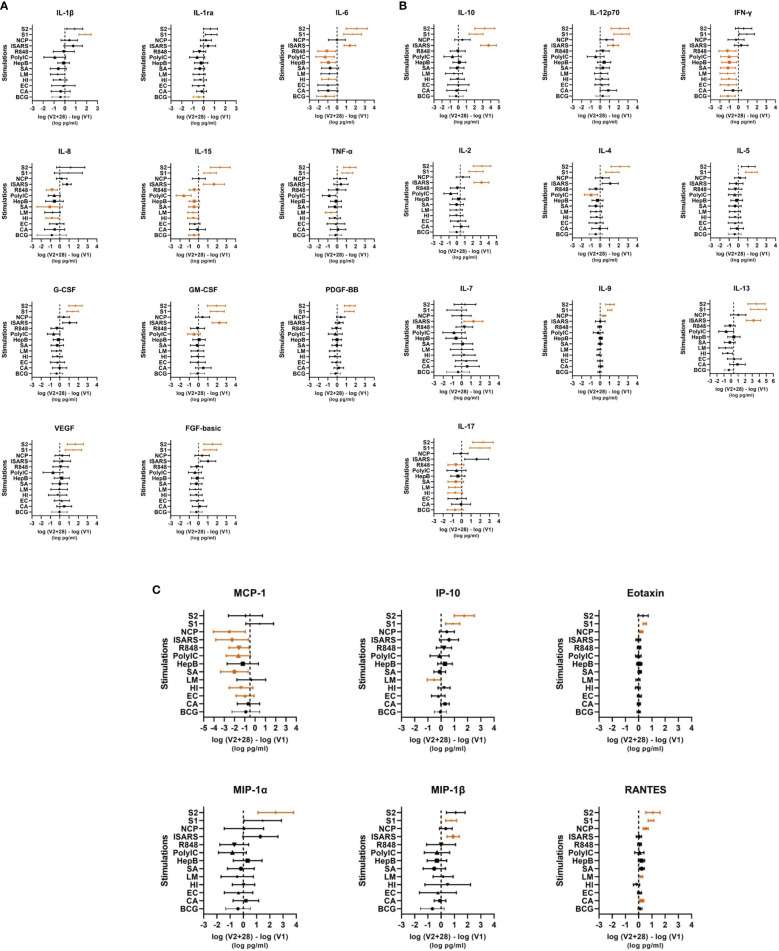
Individual cytokine responses to whole‐blood stimulations 28 days after BNT162b2 vaccination. Cytokines are grouped in **(A)** innate/inflammatory, **(B)** adaptive/mixed and **(C)** chemokine categories. The mean ± 95% confidence interval of the log differences (log(V2 + 28) – log(V1)) for each stimulation (bacille Calmette–Guérin vaccine (BCG), *C. albicans* (CA) *E. coli* (EC), *H. influenzae* (HI), *L. monocytogenes* (LM), and *S. aureus* (SA), irradiated-SARS-CoV-2 (iSARS), SARS-CoV-2 nucleocapsid protein (NCP), SARS-CoV-2 spike protein S1 (S1) and S2 (S2), hepatitis B antigen (HepB), polycytidylic acid (Poly(I:C)), and R848) are represented in each cytokine plot. Significant results p < 0.05 are depicted in orange.

Comparing cytokine responses between V1 and V2 + 28 showed that BNT162b2 vaccination resulted in increased response to iSARS, S1, and S2 stimulations for several innate, inflammatory, and adaptive cytokines, as well as some chemokines (**Figures 2**, **3**). The cytokines with the largest increases were IL-6, IL-15, GM-CSF, IL-10, IL-12p70, IL-2, and IL-13, as well as chemokines MIP-1β and RANTES. Changes in S1 and S2 stimulation responses were greater following BNT162b2 vaccination, with increased cytokines responses also observed for TNF-α, G-CSF, PDGF-BB, VEGF, FGF-basic, IL-4, IL-17, and IP-10. BNT162b2 vaccination largely did not alter cytokine responses to NCP stimulation, although there were increases in IL-9, Eotaxin, and RANTES. For SARS-CoV-2-specific stimuli, MCP-1 was the only analyte that decreased following BNT162b2 vaccination, and this occurred in response to iSARS and NCP stimulation.

Following heterologous bacterial, fungal and viral/TLR agonists stimulation, there was a general decrease in cytokine and chemokine responses in children between V1 and V2 + 28. The largest decreases were seen for IFN-γ and MCP-1 (**Figures 2**, **3A-C**). IL-6, IL-15, IL-17 also decreased between V1 and V2 + 28 following stimulation with BCG, *H. influenzae*, *S. aureus*, hepatitis B antigen, poly(I:C), and R848 (**Figure 3B**). *L. monocytogenes* stimulation induced IL-15, TNF-α and IP-10 decreases between V1 and V2 + 28 (**Figure 3C**). IL-8 responses also decreased between V1 and V2 + 28 following *H. influenzae* and *S. aureus* stimulation. RANTES was the only analyte that increased in response to heterologous stimulants (*L. monocytogenes* and *C. albicans*) between V1 and V2 + 28. A sensitivity analysis of only participants who did not receive BCG vaccination in MIS BAIR indicated that BCG status had a negligible or no effect on the reported effect of BNT162b2 vaccination on cytokine responses (**Supplementary Figure S3**).

### BNT162b2 vaccination is associated with a sustained decrease in cytokine responses to viral, but not bacterial, stimulants six months after vaccination

**Figure 4:**
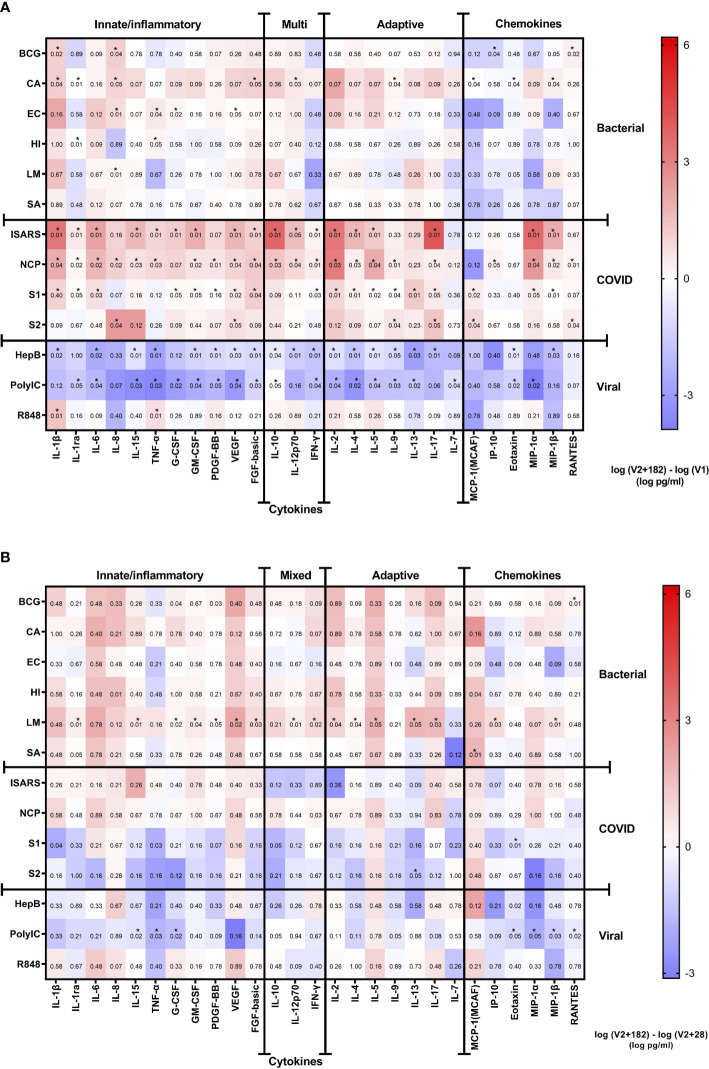
Cytokine responses to whole‐blood stimulations 182 days after BNT162b2 vaccination. Heatmaps representing **(A)** the V1 and V2 + 182 cytokine differences and **(B)** the V2 + 28 and V2 + 182 cytokine differences. The medians of 8 study participants are plotted in the heatmap colour gradient, with red representing increases and blue indicating decreases. Each row represents a stimulation condition, and is separated into three groups: bacterial/fungal stimulations (bacille Calmette-Guérin vaccine (BCG), *C. albicans* (CA), *E. coli* (EC), *H. influenzae* (HI), *L. monocytogenes* (LM), and *S. aureus* (SA)); SARS-CoV-2 stimulations (irradiated-SARS-CoV-2 (iSARS), SARS-CoV-2 nucleocapsid protein (NCP), SARS-CoV-2 spike Protein S1 (S1) and S2 (S2); and viral/TLR stimulations (hepatitis B antigen (HepB), polycytidylic acid (Poly(I:C)), resiquimod (R848)). Differences were assessed for statistical significance by the Wilcoxon signed-rank test of the difference in the logarithm of V1 and logarithm of V2 + 182 cytokine concentrations. Asterisks in the boxes depict significance (*p<0.05).

The eight children who had paired samples from V1 and V2 + 182, and who remained NCP negative were included in this analysis. There were modest changes at V2 + 182 compared to V1 (**Figure 4**). Stimulation with *C. albicans* altered several cytokines at V2 + 182 compared to V1 with increased IL-1β, IL-1ra, IL-8, FGF-basic, IL-12p70, IL-8, MIP-1 and decreased MCP-1 and Eotaxin responses. IL-8 was also increased in response to several bacterial stimulations, including BCG, *E. coli* and *H. influenzae* at V2 + 182 compared to V1 (**Figure 4**). The largest increases were in IL-1β and IL-8 cytokine at V2 + 182 compared to V1 (**Figure 4A**). Between V1 and V2 + 182, BCG stimulation-induced responses of RANTES increased, while IP-10 decreased. In addition, *E. coli* and *H. influenzae* stimulation TNF-α responses increased at V2 + 182 compared to V1. Other modest changes included increases in IL-1ra and G-CSF following *H. influenzae* and *E. coli* stimulations at V2 + 182 compared to V1.

For viral/TLR agonists (hepatitis B antigen, poly(I:C), R848) stimulations, there were decreases in several cytokine and chemokine responses in children at V2 + 182 compared to V1. Hepatitis B antigen and poly(I:C) stimulation responses were decreased for IL-6, IL-15, TNF-α, GM-CSF, PDGF-BB, VEGF, FGF-basic, IL-10, IFN-γ, IL-2, IL-4, IL-5, IL-9, IL-13, and Eotaxin at V2 + 182 compared to V1. In addition, at V2 + 182 compared to V1, hepatitis B antigen stimulation responses also decreased for IL-1β, IL-12p70, IL-17, and MIP-1β. Poly(I:C) stimulation response to IL-1ra, IL-7, and MIP-1α were also decreased at V2 + 182 compared to V1. Comparison of V2 + 182 to V1 did not show any decrease in cytokine responses to R848 stimulation except for a marked increase in IL-1β (**Figure 4A**).

Analysis of responses to SARS-CoV-2-specific stimulations showed that many of the cytokine responses were sustained from V1 to V2 + 182. Comparison of V2 + 182 to V1 showed increased iSARS and S1 stimulation responses for a range of cytokines and chemokines including IL-1β, IL-1ra, IL-6, G-CSF, GM-CSF, VEGF, FGF-basic, IFN-γ, IL-2, IL4, IL-5, IL-17, MIP-1α and MIP-1β. NCP stimulation responses for a wide range of cytokines were altered between V1 and V2 + 182, with increases in 22/27 cytokine and chemokine analytes (**Figure 4A**). Fewer cytokines maintained increased responses to S2 stimulation between V2 + 182 and V1 (5 at V2 + 182 compared to 17 at V2 + 28).

For 8 of 14 stimulations, there were no changes in cytokine response from V2 + 28 to V2 + 182 in the 8 participants with paired samples at V1, V2 + 28 and V2 + 182. Although only IL-8 was significantly increased from V1 to V2 + 182 following *L. monocytogenes* stimulation (**Figure 4A**), at V2 + 28 to V2 + 182 there were increases in 16 out of 27 cytokines/chemokines (**Figure 4B**). Poly(I:C) stimulation responses were decreased for 7 out of 27 cytokines between V2 + 28 and V2 + 182.

### SARS-CoV-2-specific immunity did not correlate with heterologous cytokine responses

**Figure 5:**
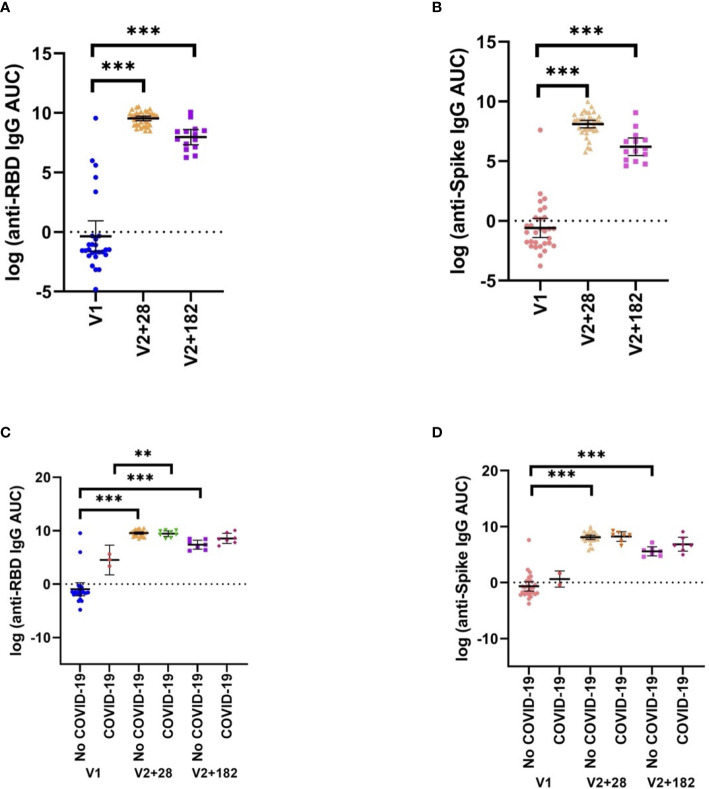
SARS-CoV-2-specific adaptive immunity. **(A)** Anti-RBD, and **(B)** anti-spike IgG antibody titres pre- (V1) and post- (V2 + 28, V2 + 182) BNT162b2 vaccination of all participants with matched samples (V1, n=37; V2, n=37; V2 + 182, n=14). These data are separated by previous COVID-19 status by V1, V2 + 28, and V2 + 182 in **(C)** anti-RBD and **(D)** anti-spike (No COVID-19: V1, n=35; V2 + 28, n=29, V3 + 182, n=7; COVID-19: V1, n=2; V2 + 28, n=8, V3 + 182, n=7). Differences were assessed for statistical significance using paired t-tests (**p<0.01, ***p<0.001).

Anti-spike and anti-RBD total IgG antibody titres were measured at V1, V2 + 28 and V2 + 182 for all participants. All participants with paired samples (n=38) had robust anti-spike and anti-RBD IgG antibody titres 28 days following the second dose of BNT162b2 vaccination (**Figures 5A, B**). Anti-spike/RBD IgG antibody titre increases were sustained at V2 + 182. Prior COVID-19 did not significantly increase anti-spike or anti-RBD IgG antibody titre before or after vaccination (**Figures 5C, D**). Anti-spike IgG antibody titre and anti-RBD IgG antibody titre were positively correlated at V2 + 28 (data not shown). Anti-RBD IgG antibody titre and heterologous stimulation cytokine response largely did not correlate. That is, there were few correlations between V1 to V2 + 28 differences in anti-RBD IgG antibody titre and the V1 to V2 + 28 difference in cytokine responses to SARS-CoV-2 or heterologous stimulants (**Supplementary Table S3**).

## Discussion

The main findings of our study in children are that: (i) BNT162b2 vaccination alters heterologous bacterial and viral cytokine responses 28 and 182 days after the primary vaccination schedule, compared to pre-vaccination; (ii) the effect of BNT162b2 vaccination on heterologous immunity persists for viral but not bacterial stimulants; and (iii) there is no correlation between the heterologous immunological effects and vaccine-specific IgG responses to BNT162b2. Our study provides unique information regarding the heterologous effects of COVID-19 vaccination in a paediatric population.

Our study showed that, in children, SARS-CoV-2 mRNA vaccination decreases inflammatory cytokine responses (IFN-γ, MCP-1, IL-6, IL-8 and IL-15) to heterologous bacterial, fungal and viral re-stimulation. A pre-print of a study in 16 adult healthcare workers reported heterologous effects of the same mRNA-based vaccine used in our study (26). The study in adults found decreases in IFN-γ and IL-6 production after stimulation with heterologous stimulants of bacterial and viral origin and increases in inflammatory cytokine production after *C. albicans* stimulation (26), in keeping with our findings. Using a replication-deficient adenovirus COVID-19 vaccine, Murphy et al. showed contrasting results: ChAdOx1 nCoV-19 vaccination of 10 adult volunteers was associated with increased IL-6, MCP-1 and IFN-γ production after TLR (LPS or Pam3Csk4) and mycobacterial (*M. tuberculosis*) stimulation (20). The inconsistencies between our findings and that of Murphy et al. may reflect the difference in vaccine platform or age of participants.

SARS-CoV-2 mRNA vaccination in mice and humans resulted in enhanced innate immune responses, including enhanced plasma IFN-γ concentrations after the second vaccination (21, 27). The RIG-I/MDA5–IFNAR1 signalling pathway is essential for IFN-γ and other cytokine (such as IL-6, IFN-α, MCP-1 and MIP-1β) production, and innate and adaptive cell activation after the BNT162b2 vaccine in mice (27). The activation of this pathway results in interference between cytosolic RIG-I-like receptor and membrane-bound Toll-like receptor signalling (28–30), and increased susceptibility to respiratory disease in viral and bacterial coinfections (28). A possible mechanism for the decreased heterologous cytokine responses after BNT162b2 vaccination observed in our study is interference by the BNT162b2-induced RIG-I/MDA5-IFNAR1 pathway on pattern recognition receptor-mediated responses to heterologous ligands. COVID-19 mRNA-based vaccines have been shown to modulate transcriptional profiles in innate immune cells, generating a unique mixture of myeloid cells (21, 27), cells which have been associated with enhanced resistance against heterologous viruses (31). Our results add to the evolving evidence that SARS-CoV-2 mRNA vaccination reprograms both adaptive and innate immune responses.

We showed evidence of temporal associations between BNT162b2 vaccination and altered heterologous effects. We hypothesised that another way to demonstrate this association might be by showing a ‘dose-response’ relationship between BNT162b2 vaccination and altered heterologous effects. This was the rationale behind performing the correlation analyses between anti-RBD IgG antibody titre and heterologous stimulation cytokine response. There was no consistent correlation between BNT162b2 vaccination-induced anti-RBD IgG antibody titre at V2 + 28 and cytokine responses. This may suggest that the mechanisms driving these responses may not be directly interconnected.

It has been postulated that COVID-19 vaccines that provide a combinatorial effect from both adaptive and trained immunity may result in more potent and broader protection (32, 33). Our findings suggest SARS-CoV-2 mRNA vaccination could alter the immune response to other pathogens, which cause both vaccine-preventable and non-vaccine-preventable diseases (34, 35). This is particularly relevant in children as they: have extensive exposure to microbes at daycare, school, and social occasions; are often encountering these microbes for the first time; and receive multiple vaccines as part of routine childhood vaccination schedules. There are currently no data on the clinical effects of COVID-19 vaccination-related heterologous effects in children.

The strengths of this study include that it is the first to assess the heterologous effects of a COVID-19 vaccine in children, the participants are from a well-described cohort followed up since birth, and the use of longitudinal follow-up with multiple sampling. Community transmission of SARS-CoV-2 was low prior to January 2022 in Melbourne; therefore, children were unlikely to have prior SARS-CoV-2 infections and/or waned NCP antibody responses. NCP antibody reactivity allowed the exclusion of children with previous SARS-CoV-2 infection, instead of relying on parent-reported infection or symptomatic diaries. The use of many stimulants and the measurement of multiple cytokines allowed a broad examination of the immune response. Further strengths are the standardised procedures used for blood collection, handling of samples and laboratory assays (36) with which we have considerable experience.

Limitations include the inability to include an unvaccinated control group due to the ATAGI recommendation for all children aged 5 to 11 years to receive the BNT162b2 vaccine. It was unethical to randomise children into an unvaccinated, placebo or delayed vaccination group, given that ATAGI recommended the BNT162b2 vaccine for the age group of interest and that Melbourne was experiencing a surge in COVID-19 cases in the community during the study period. As a pragmatic solution we designed the trial to use each child as their own comparator, before and after vaccination. The large proportion of parents not willing to return for the optional visit at V2 + 182 led to a reduced number of samples available for analyses at this time point. The participants of this trial were a sample from a larger randomised-control trial in a high-income setting. The generalisability of our results might be affected by the demographics of the participants in the MIS BAIR randomised-control trial from which participants were drawn; for example, a large proportion had a family history of allergic and atopic disease (37). Correction for multiple comparisons was not done as this was an exploratory analysis that aimed to find consistent patterns in mRNA vaccine-induced alterations in cytokine responses and heterologous immunity. We have shown all analyses in their entirety to assist interpretation of the results.

These data show that a SARS-CoV-2 mRNA-based vaccine alters heterologous immunity in children and that these effects can persist up to six months after vaccination. Whether SARS-CoV-2 mRNA-based vaccines can induce the epigenetic and metabolic changes associated with trained immunity to provide protection against other infectious diseases remains an open question. That SARS-CoV-2 mRNA vaccination in children could impact immune responses to other pathogens emphasises the need for further research and consideration of heterologous effects in vaccination policies given their broad public health implications.

## Data availability statement

The raw data supporting the conclusions of this article will be made available by the authors, without undue reservation.

## Ethics statement

The studies involving humans were approved by Royal Children’s Hospital, Melbourne (HREC/81771/RCHM-2021). The studies were conducted in accordance with the local legislation and institutional requirements. Written informed consent for participation in this study was provided by the participants’ legal guardians/next of kin.

## Author contributions

AN, DB, KP, NC, NM conceived the study. AN, CA, EB, SE conducted participant recruitment, screening and vaccination, and sample collection. AN, TD, SG performed the laboratory and statistical analyses. AN, TD wrote the manuscript. AN, TD, DB, KP, NC, NM critically reviewed the manuscript. All authors contributed to the article and approved the submitted version.

## Supplementary material

Supplementary Figure 1 Study timeline. Participants were requested to provide blood samples at two core visits and one optional visit. The first blood sample was taken just before, and on the same day, as the first BNT162b2 vaccination (V1), the second blood sample was taken 28 days after the second BNT162b2 vaccination (V2 + 28) and the optional third blood sample was taken 6 months after the second BNT162b2 vaccination (V2 + 182). The needle represents vaccination with BNT162b2, and the drops represent blood sampling. Time is indicated in days.

Supplementary Figure 2 Individual cytokine and chemokine concentrations. Cytokines are grouped in **(A)** innate, **(B)** growth factor, **(C)** mixed, **(D)** adaptive and **(E)** chemokine categories. Logarithm transformed cytokine and chemokine responses to bacille Calmette-Guérin vaccine (BCG), *C. albicans* (CA) *E. coli* (EC), *H. influenzae* (HI), *L. monocytogenes* (LM), *S. aureus* (SA), irradiated-SARS-CoV-2 (iSARS), SARS-CoV-2 nucleocapsid protein (NCP), SARS-CoV-2 spike Protein S1 (S1) and S2 (S2), hepatitis B antigen (HepB), polycytidylic acid (Poly(I:C)), and resiquimod (R848) at all timepoints.

Supplementary Figure 3 BCG sensitivity analysis of cytokine responses to whole‐blood stimulations 28 days after BNT162b2 vaccination. The mean of the pre- (V1) and post- (V2 + 28) cytokine differences from 25 study participants with no BCG vaccination is plotted in the heatmap colour gradient, with red representing increases and blue indicating decreases. Each row represents a stimulation condition, and is separated into three groups: bacterial/fungal stimulations (bacille Calmette-Guérin vaccine (BCG), *C. albicans* (CA), *E. coli* (EC), *H. influenzae* (HI), *L. monocytogenes* (LM), and *S. aureus* (SA)); SARS-CoV-2 stimulations (irradiated-SARS-CoV-2 (iSARS), SARS-CoV-2 nucleocapsid protein (NCP), SARS-CoV-2 spike Protein S1 (S1) and S2 (S2); and viral/TLR stimulations (hepatitis B antigen (HepB), polycytidylic acid (Poly(I:C)), resiquimod (R848)). Differences were assessed for statistical significance by paired t-tests of the difference in the logarithm of V1 and logarithm of V2 + 28 cytokine concentrations, p-values for each test are represented in respective boxes. Asterisks in the boxes depict significance (*p<0.05, **p<0.01 and ***p<0.001).
