# Supplementary material for: BNT162b2 COVID-19 vaccination in children alters cytokine responses to heterologous pathogens and Toll-like receptor agonists
Source: Front Immunol. 2023 Aug 25;14:1242380. doi: 10.3389/fimmu.2023.1242380 (PMC10485613; doi:10.3389/fimmu.2023.1242380)
Supplement: Supplementary file 1 [file Table_1.docx]

**Supplementary table 1.** Proportion of stimulations above the limit of detection. The following cytokines were all within the detectable limit of the assay: Eotaxin, Basic FGF, G‐CSF, GM‐CSF, IFN‐γ, IL‐1β, IL‐1rα, IL‐2, IL‐4, IL‐5, IL‐7, IL‐8, IL‐9, IL‐10, IL‐12(p70), IL‐13, IL‐15, IL‐17, IP‐10, MIP‐1β, PDGF‐BB, and RANTES.

|  | **IL-6** | **MCP-1** | **MIP-1α** | **TNF-α** | **VEGF** |
| --- | --- | --- | --- | --- | --- |
| **V1** | 0.53% | 6.06% | 6.60% | 0.18% | 0.18% |
| **V2+28** | 1.07% | 11.59% | 8.38% | 0.00% | 0.00% |

**Supplementary table 2.** Standardisation of study procedures.

|  | V1  (mean ± SD) *hours*  n=47 | V2+28  (mean ± SD) *hours*  n=38 | V2+182  (mean ± SD) *hours*  n=14 |
| --- | --- | --- | --- |
| Time between blood collection and stimulation | 2.1 ± 0.4 | 2.0 ± 0.1 | 2.0 ± 0.2 |
| Time of stimulation incubation | 21.1 ± 1.3 | 20.1 ± 0.9 | 20.2 ± 1 |

|  | V1 to V2  (median (Q1-Q3)) *days*  n=45 | V2 to V2+28  (median (Q1-Q3)) *days*  n=38 | V2 to V2+182  (median (Q1-Q3)) *days*  n=15 |
| --- | --- | --- | --- |
| Time between time points | 56 (56-58) | 27 (25-29) | 187 (181-192) |

|  | V1  n=47 | V2+28  n=38 | V2+182  n=14 |
| --- | --- | --- | --- |
| Age at first blood collection  (median (Q1-Q3)) (years) | 6.4 (5.9-6.8) |  |  |
| Volume of blood (mean ± SD) (mL) | 21.2 ± 4.4 | 22.8 ± 0.5 | 22.5 ± 0.9 |
| Stimulation within  4 (± 0.4) hours (n (%)) | 39 (83.0%) | 36 (94.7%) | 15 (100%) |
| Incubation for  20 (± 2) hours (n (%)) | 38 (80.9%) | 38 (100%) | 15 (100%) |

| **Supplementary table 3.** | Spearman's rank correlation coefficient of RBD IgG antibody titre log(AUC) vs cytokine response (log(V2+28) - log(V1)). Highlighted red boxes indicate a p-value <0.05. | | | | | | | |  |  |  |
| --- | --- | --- | --- | --- | --- | --- | --- | --- | --- | --- | --- |
|  |  |  |  |  |  |  |  |  |  |  |  |
| **iSARS stim** | | | **S1 stim** | | | **S2 stim** | | | **NCP stim** | | |
| **Cytokines** | **correlation coefficient** | **p-value** | **Cytokines** | **correlation coefficient** | **p-value** | **Cytokines** | **correlation coefficient** | **p-value** | **Cytokines** | **correlation coefficient** | **p-value** |
| IL-1β | -0.05 | 0.85 | IL-1β | 0.12 | 0.65 | IL-1β | -0.12 | 0.65 | IL-1β | 0.108 | 0.68 |
| IL-ra | -0.05 | 0.85 | IL-ra | 0.12 | 0.65 | IL-ra | -0.12 | 0.65 | IL-ra | -0.15 | 0.56 |
| IL-2 | 0.23 | 0.28 | IL-2 | 0.45 | 0.07 | IL-2 | 0.28 | 0.28 | IL-2 | 0.39 | 0.12 |
| IL-4 | -0.08 | 0.77 | IL-4 | 0.15 | 0.55 | IL-4 | -0.04 | 0.87 | IL-4 | -0.09 | 0.74 |
| IL-5 | -0.23 | 0.24 | IL-5 | 0.35 | 0.17 | IL-5 | 0.26 | 0.32 | IL-5 | 0.27 | 0.31 |
| IL-6 | -0.44 | 0.08 | IL-6 | 0.06 | 0.82 | IL-6 | -0.15 | 0.57 | IL-6 | -0.11 | 0.69 |
| IL-7 | 0.13 | 0.63 | IL-7 | 0.06 | 0.83 | IL-7 | -0.03 | 0.90 | IL-7 | 0.21 | 0.42 |
| IL-8 | 0.01 | 0.96 | IL-8 | 0.24 | 0.36 | IL-8 | -0.12 | 0.66 | IL-8 | 0.37 | 0.14 |
| IL-9 | 0.10 | 0.70 | IL-9 | 0.23 | 0.37 | IL-9 | -0.03 | 0.91 | IL-9 | -0.06 | 0.82 |
| IL-10 | -0.52 | 0.03 | IL-10 | -0.29 | 0.26 | IL-10 | -0.42 | 0.10 | IL-10 | -0.38 | 0.14 |
| IL-12p70 | -0.05 | 0.84 | IL-12p70 | 0.30 | 0.24 | IL-12p70 | 0.21 | 0.21 | IL-12p70 | 0.38 | 0.14 |
| IL-13 | 0.30 | 0.23 | IL-13 | -0.10 | 0.71 | IL-13 | -0.14 | 0.59 | IL-13 | -0.46 | 0.06 |
| IL-15 | -0.30 | 0.12 | IL-15 | 0.21 | 0.42 | IL-15 | 0.09 | 0.74 | IL-15 | 0.14 | 0.60 |
| IL-17 | 0.20 | 0.45 | IL-17 | 0.32 | 0.21 | IL-17 | 0.32 | 0.21 | IL-17 | -0.10 | 0.72 |
| Eotaxin | -0.26 | 0.30 | Eotaxin | -0.22 | 0.40 | Eotaxin | -0.20 | 0.44 | Eotaxin | -0.24 | 0.35 |
| FGF-basic | 0.01 | 0.97 | FGF-basic | 0.14 | 0.61 | FGF-basic | 0.06 | 0.81 | FGF-basic | 0.03 | 0.92 |
| G-CSF | 0.04 | 0.88 | G-CSF | 0.40 | 0.11 | G-CSF | 0.38 | 0.38 | G-CSF | 0.21 | 0.41 |
| GM-CSF | 0.22 | 0.41 | GM-CSF | 0.28 | 0.29 | GM-CSF | -0.01 | 0.99 | GM-CSF | 0.11 | 0.67 |
| IFN-γ | 0.09 | 0.74 | IFN-γ | 0.40 | 0.11 | IFN-γ | 0.30 | 0.24 | IFN-γ | 0.18 | 0.50 |
| MCP-1 | 0.04 | 0.87 | MCP-1 | 0.23 | 0.37 | MCP-1 | 0.24 | 0.35 | MCP-1 | 0.35 | 0.17 |
| IP10 | -0.20 | 0.45 | IP10 | -0.05 | 0.86 | IP10 | -0.11 | 0.69 | IP10 | -0.44 | 0.08 |
| PDGF-BB | 0.17 | 0.52 | PDGF-BB | 0.06 | 0.81 | PDGF-BB | -0.03 | 0.93 | PDGF-BB | 0.00 | 1.00 |
| MIP-1α | -0.16 | 0.53 | MIP-1α | 0.32 | 0.21 | MIP-1α | 0.26 | 0.31 | MIP-1α | 0.23 | 0.38 |
| MIP-1β | -0.01 | 0.99 | MIP-1β | 0.15 | 0.58 | MIP-1β | 0.05 | 0.85 | MIP-1β | -0.28 | 0.28 |
| RANTES | 0.27 | 0.29 | RANTES | 0.05 | 0.85 | RANTES | -0.01 | 0.96 | RANTES | 0.24 | 0.36 |
| TNF-α | -0.09 | 0.72 | TNF-α | 0.23 | 0.38 | TNF-α | 0.04 | 0.87 | TNF-α | -0.07 | 0.80 |
| VEGF | -0.16 | 0.54 | VEGF | 0.26 | 0.02 | VEGF | 0.38 | 0.14 | VEGF | 0.51 | 0.04 |

| **BCG** | | | **CA** | | | **EC** | | | **HI** | | | **LM** | | | **SA** | | |
| --- | --- | --- | --- | --- | --- | --- | --- | --- | --- | --- | --- | --- | --- | --- | --- | --- | --- |
| **Cytokines** | **correlation coefficient** | **p-value** | **Cytokines** | **correlation coefficient** | **p-value** | **Cytokines** | **correlation coefficient** | **p-value** | **Cytokines** | **correlation coefficient** | **p-value** | **Cytokines** | **correlation coefficient** | **p-value** | **Cytokines** | **correlation coefficient** | **p-value** |
| IL-1β | 0.15 | 0.57 | IL-1β | 0.42 | 0.10 | IL-1β | 0.07 | 0.79 | IL-1β | 0.28 | 0.28 | IL-1β | 0.31 | 0.23 | IL-1β | 0.14 | 0.59 |
| IL-ra | -0.05 | 0.85 | IL-ra | -0.15 | 0.56 | IL-ra | 0.04 | 0.89 | IL-ra | 0.03 | 0.92 | IL-ra | 0.13 | 0.63 | IL-ra | 0.10 | 0.72 |
| IL-2 | 0.36 | 0.16 | IL-2 | 0.43 | 0.09 | IL-2 | 0.40 | 0.12 | IL-2 | 0.32 | 0.21 | IL-2 | 0.59 | 0.01 | IL-2 | 0.44 | 0.08 |
| IL-4 | 0.00 | 0.99 | IL-4 | 0.24 | 0.36 | IL-4 | 0.03 | 0.92 | IL-4 | 0.05 | 0.85 | IL-4 | 0.25 | 0.33 | IL-4 | 0.16 | 0.55 |
| IL-5 | 0.33 | 0.20 | IL-5 | 0.36 | 0.16 | IL-5 | 0.30 | 0.25 | IL-5 | 0.32 | 0.21 | IL-5 | 0.46 | 0.06 | IL-5 | 0.29 | 0.26 |
| IL-6 | 0.07 | 0.80 | IL-6 | 0.09 | 0.74 | IL-6 | 0.08 | 0.76 | IL-6 | 0.14 | 0.59 | IL-6 | 0.07 | 0.78 | IL-6 | 0.10 | 0.72 |
| IL-7 | 0.37 | 0.14 | IL-7 | -0.07 | 0.79 | IL-7 | 0.02 | 0.93 | IL-7 | 0.36 | 0.15 | IL-7 | 0.33 | 0.19 | IL-7 | -0.05 | 0.84 |
| IL-8 | 0.49 | 0.05 | IL-8 | 0.21 | 0.43 | IL-8 | 0.28 | 0.27 | IL-8 | 0.29 | 0.26 | IL-8 | 0.53 | 0.03 | IL-8 | 0.43 | 0.09 |
| IL-9 | 0.00 | 1.00 | IL-9 | 0.19 | 0.47 | IL-9 | 0.02 | 0.94 | IL-9 | 0.04 | 0.87 | IL-9 | 0.08 | 0.75 | IL-9 | 0.06 | 0.82 |
| IL-10 | -0.03 | 0.91 | IL-10 | 0.21 | 0.42 | IL-10 | -0.14 | 0.59 | IL-10 | -0.24 | 0.35 | IL-10 | 0.01 | 0.97 | IL-10 | -0.22 | 0.39 |
| IL-12p70 | 0.39 | 0.13 | IL-12p70 | 0.29 | 0.26 | IL-12p70 | 0.28 | 0.27 | IL-12p70 | 0.52 | 0.03 | IL-12p70 | 0.36 | 0.16 | IL-12p70 | 0.41 | 0.10 |
| IL-13 | -0.26 | 0.35 | IL-13 | -0.36 | 0.16 | IL-13 | -0.33 | 0.20 | IL-13 | -0.38 | 0.14 | IL-13 | 0.34 | 0.18 | IL-13 | 0.05 | 0.84 |
| IL-15 | 0.15 | 0.57 | IL-15 | 0.34 | 0.18 | IL-15 | 0.41 | 0.10 | IL-15 | 0.24 | 0.36 | IL-15 | 0.52 | 0.03 | IL-15 | 0.30 | 0.24 |
| IL-17 | 0.37 | 0.15 | IL-17 | 0.34 | 0.18 | IL-17 | 0.30 | 0.24 | IL-17 | 0.32 | 0.21 | IL-17 | 0.40 | 0.11 | IL-17 | 0.27 | 0.30 |
| Eotaxin | -0.33 | 0.20 | Eotaxin | 0.33 | 0.20 | Eotaxin | -0.20 | 0.43 | Eotaxin | -0.04 | 0.88 | Eotaxin | -0.04 | 0.89 | Eotaxin | -0.24 | 0.36 |
| FGF-basic | 0.19 | 0.46 | FGF-basic | 0.20 | 0.44 | FGF-basic | 0.10 | 0.72 | FGF-basic | 0.18 | 0.49 | FGF-basic | 0.23 | 0.37 | FGF-basic | 0.14 | 0.59 |
| G-CSF | 0.36 | 0.16 | G-CSF | 0.50 | 0.04 | G-CSF | 0.28 | 0.27 | G-CSF | 0.38 | 0.14 | G-CSF | 0.49 | 0.05 | G-CSF | 0.46 | 0.06 |
| GM-CSF | 0.11 | 0.68 | GM-CSF | 0.10 | 0.69 | GM-CSF | 0.27 | 0.30 | GM-CSF | 0.14 | 0.59 | GM-CSF | 0.30 | 0.24 | GM-CSF | 0.18 | 0.48 |
| IFN-γ | 0.42 | 0.09 | IFN-γ | 0.31 | 0.22 | IFN-γ | 0.31 | 0.23 | IFN-γ | 0.33 | 0.94 | IFN-γ | 0.40 | 0.12 | IFN-γ | 0.40 | 0.11 |
| MCP-1 | -0.22 | 0.39 | MCP-1 | 0.25 | 0.34 | MCP-1 | 0.27 | 0.31 | MCP-1 | -0.16 | 0.20 | MCP-1 | 0.00 | 1.00 | MCP-1 | 0.55 | 0.02 |
| IP10 | -0.03 | 0.91 | IP10 | -0.28 | 0.27 | IP10 | -0.14 | 0.59 | IP10 | -0.17 | 0.54 | IP10 | 0.00 | 1.00 | IP10 | -0.11 | 0.69 |
| PDGF-BB | -0.04 | 0.87 | PDGF-BB | 0.14 | 0.59 | PDGF-BB | -0.06 | 0.83 | PDGF-BB | 0.02 | 0.52 | PDGF-BB | 0.13 | 0.62 | PDGF-BB | 0.07 | 0.78 |
| MIP-1α | 0.36 | 0.15 | MIP-1α | 0.43 | 0.09 | MIP-1α | 0.12 | 0.65 | MIP-1α | 0.33 | 0.94 | MIP-1α | 0.06 | 0.42 | MIP-1α | 0.30 | 0.24 |
| MIP-1β | 0.14 | 0.59 | MIP-1β | 0.27 | 0.29 | MIP-1β | 0.37 | 0.15 | MIP-1β | 0.09 | 0.72 | MIP-1β | 0.03 | 0.83 | MIP-1β | 0.15 | 0.57 |
| RANTES | -0.21 | 0.43 | RANTES | 0.05 | 0.85 | RANTES | 0.07 | 0.80 | RANTES | 0.00 | 0.99 | RANTES | 0.21 | 0.90 | RANTES | 0.00 | 0.99 |
| TNF-α | 0.15 | 0.57 | TNF-α | 0.17 | 0.50 | TNF-α | -0.06 | 0.83 | TNF-α | 0.02 | 0.93 | TNF-α | 0.69 | 0.42 | TNF-α | 0.22 | 0.40 |
| VEGF | 0.62 | 0.01 | VEGF | 0.51 | 0.04 | VEGF | 0.57 | 0.02 | VEGF | 0.67 | 0.00 | VEGF | 0.14 | 0.00 | VEGF | 0.62 | 0.01 |

| **R848** | | | **Poly(I:C)** | | | **HepB** | | |
| --- | --- | --- | --- | --- | --- | --- | --- | --- |
| **Cytokines** | **correlation coefficient** | **p-value** | **Cytokines** | **correlation coefficient** | **p-value** | **Cytokines** | **correlation coefficient** | **p-value** |
| IL-1β | 0.14 | 0.60 | IL-1β | 0.20 | 0.44 | IL-1β | 0.40 | 0.11 |
| IL-ra | 0.01 | 0.98 | IL-ra | 0.10 | 0.72 | IL-ra | -0.06 | 0.81 |
| IL-2 | 0.38 | 0.14 | IL-2 | 0.28 | 0.27 | IL-2 | 0.49 | 0.04 |
| IL-4 | 0.10 | 0.70 | IL-4 | 0.05 | 0.84 | IL-4 | 0.12 | 0.64 |
| IL-5 | 0.29 | 0.26 | IL-5 | 0.30 | 0.24 | IL-5 | 0.36 | 0.16 |
| IL-6 | 0.21 | 0.43 | IL-6 | 0.06 | 0.81 | IL-6 | 0.60 | 0.60 |
| IL-7 | 0.32 | 0.22 | IL-7 | 0.01 | 0.96 | IL-7 | 0.24 | 0.36 |
| IL-8 | 0.18 | 0.49 | IL-8 | 0.17 | 0.52 | IL-8 | 0.51 | 0.03 |
| IL-9 | 0.04 | 0.88 | IL-9 | 0.17 | 0.52 | IL-9 | 0.12 | 0.65 |
| IL-10 | -0.36 | 0.16 | IL-10 | 0.13 | 0.61 | IL-10 | -0.28 | 0.28 |
| IL-12p70 | 0.32 | 0.21 | IL-12p70 | 0.33 | 0.20 | IL-12p70 | 0.48 | 0.05 |
| IL-13 | -0.40 | 0.11 | IL-13 | 0.08 | 0.75 | IL-13 | -0.10 | 0.69 |
| IL-15 | 0.21 | 0.41 | IL-15 | 0.29 | 0.27 | IL-15 | 0.44 | 0.08 |
| IL-17 | 0.28 | 0.28 | IL-17 | 0.24 | 0.36 | IL-17 | 0.29 | 0.26 |
| Eotaxin | -0.35 | 0.17 | Eotaxin | -0.02 | 0.94 | Eotaxin | -0.05 | 0.84 |
| FGF-basic | 0.24 | 0.35 | FGF-basic | 0.14 | 0.60 | FGF-basic | 0.16 | 0.55 |
| G-CSF | 0.28 | 0.28 | G-CSF | 0.33 | 0.20 | G-CSF | 0.50 | 0.04 |
| GM-CSF | 0.24 | 0.35 | GM-CSF | 0.24 | 0.35 | GM-CSF | 0.17 | 0.51 |
| IFN-γ | 0.25 | 0.34 | IFN-γ | 0.34 | 0.18 | IFN-γ | 0.31 | 0.22 |
| MCP-1 | 0.29 | 0.26 | MCP-1 | 0.28 | 0.27 | MCP-1 | 0.20 | 0.44 |
| IP10 | -0.20 | 0.45 | IP10 | -0.26 | 0.32 | IP10 | -0.19 | 0.47 |
| PDGF-BB | -0.01 | 0.98 | PDGF-BB | -0.07 | 0.79 | PDGF-BB | 0.08 | 0.77 |
| MIP-1α | 0.18 | 0.49 | MIP-1α | 0.36 | 0.17 | MIP-1α | 0.41 | 0.11 |
| MIP-1β | 0.30 | 0.24 | MIP-1β | -0.05 | 0.85 | MIP-1β | -0.01 | 0.98 |
| RANTES | -0.11 | 0.68 | RANTES | 0.16 | 0.54 | RANTES | 0.03 | 0.92 |
| TNF-α | -0.01 | 0.98 | TNF-α | 0.15 | 0.26 | TNF-α | 0.12 | 0.65 |
| VEGF | 0.61 | 0.01 | VEGF | 0.51 | 0.04 | VEGF | 0.66 | 0.00 |
