# Supplementary figures and images for: BNT162b2 COVID-19 vaccination in children alters cytokine responses to heterologous pathogens and Toll-like receptor agonists
Source: Front Immunol. 2023 Aug 25;14:1242380. doi: 10.3389/fimmu.2023.1242380 (PMC10485613; doi:10.3389/fimmu.2023.1242380)

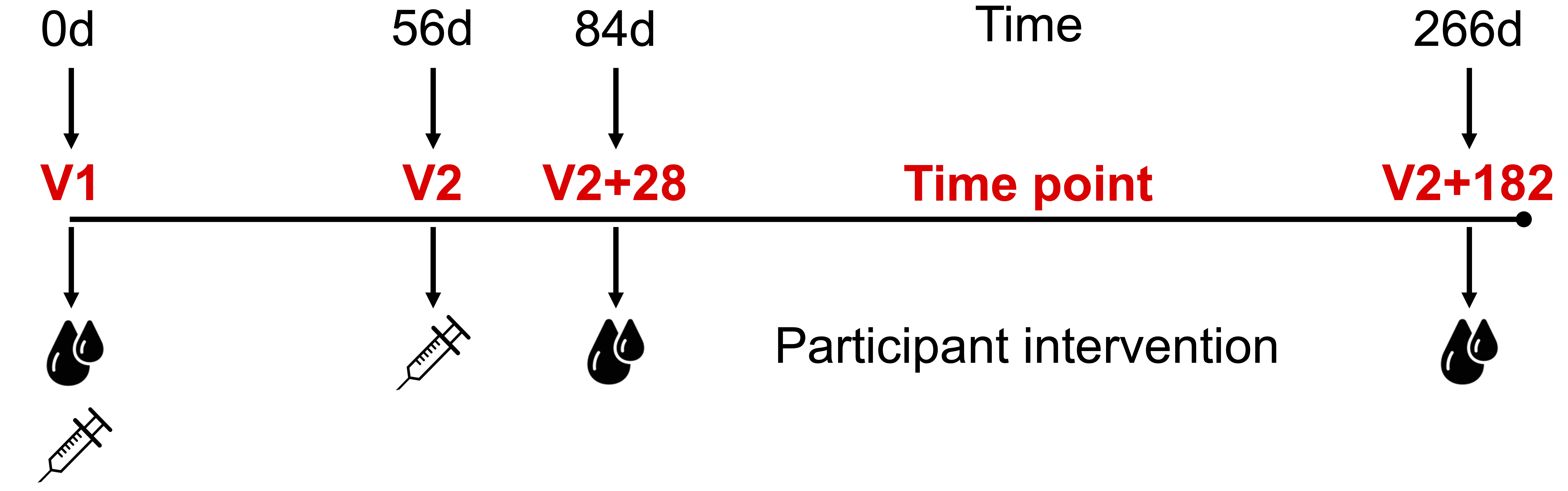

Supplement: Supplementary Figure 1 — Study timeline. Participants were requested to provide blood samples at two core visits and one optional visit. The first blood sample was taken just before, and on the same day, as the first BNT162b2 vaccination (V1), the second blood sample was taken 28 days after the second BNT162b2 vaccination (V2 + 28) and the optional third blood sample was taken 6 months after the second BNT162b2 vaccination (V2 + 182). The needle represents vaccination with BNT162b2, and the drops represent blood sampling. Time is indicated in days. [file DataSheet_1.zip › Supplementary Figure 1.jpg]

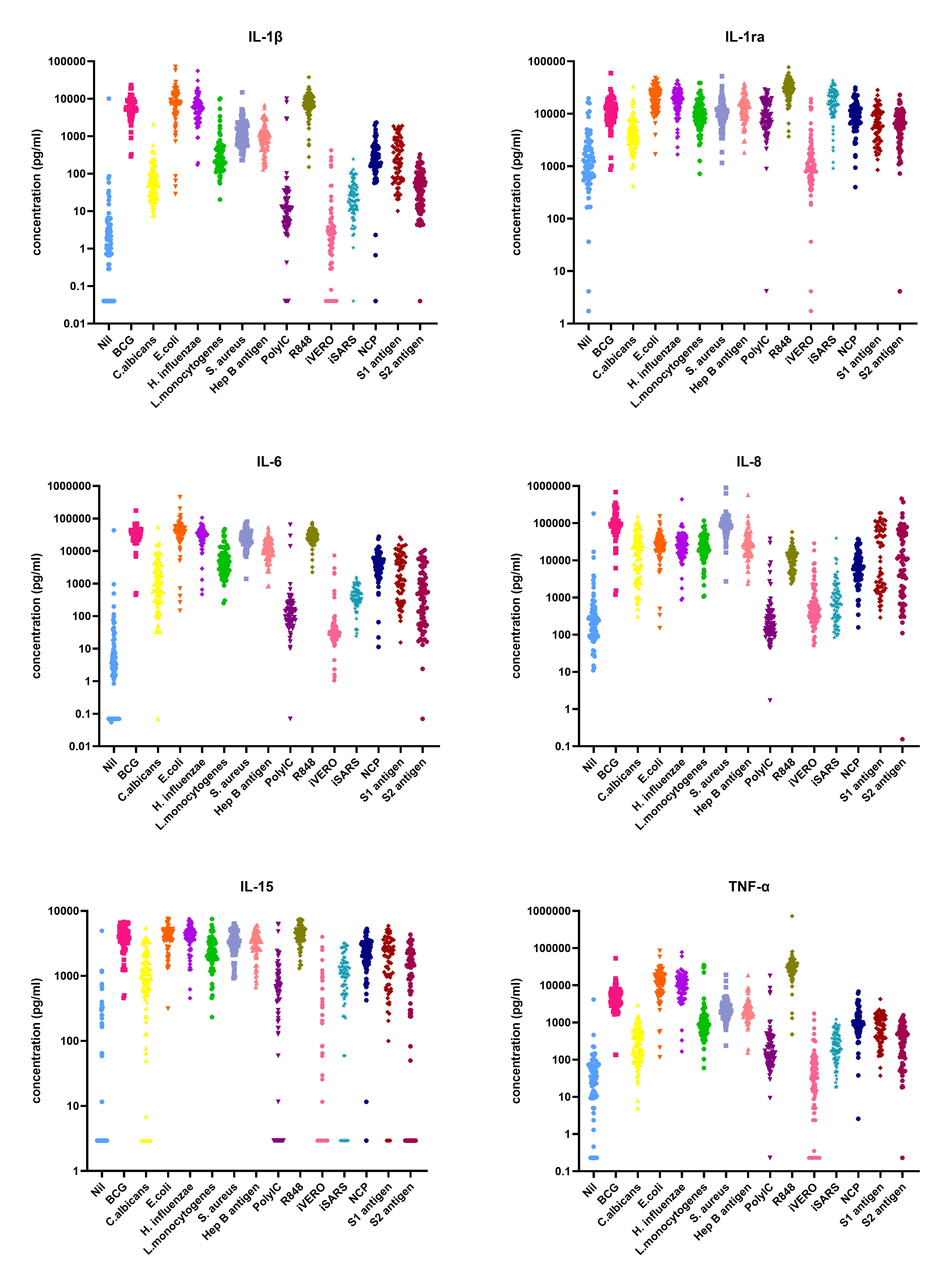

Supplement: Supplementary Figure 1 — Study timeline. Participants were requested to provide blood samples at two core visits and one optional visit. The first blood sample was taken just before, and on the same day, as the first BNT162b2 vaccination (V1), the second blood sample was taken 28 days after the second BNT162b2 vaccination (V2 + 28) and the optional third blood sample was taken 6 months after the second BNT162b2 vaccination (V2 + 182). The needle represents vaccination with BNT162b2, and the drops represent blood sampling. Time is indicated in days. [file DataSheet_1.zip › Supplementary Figure 2(A).jpg]

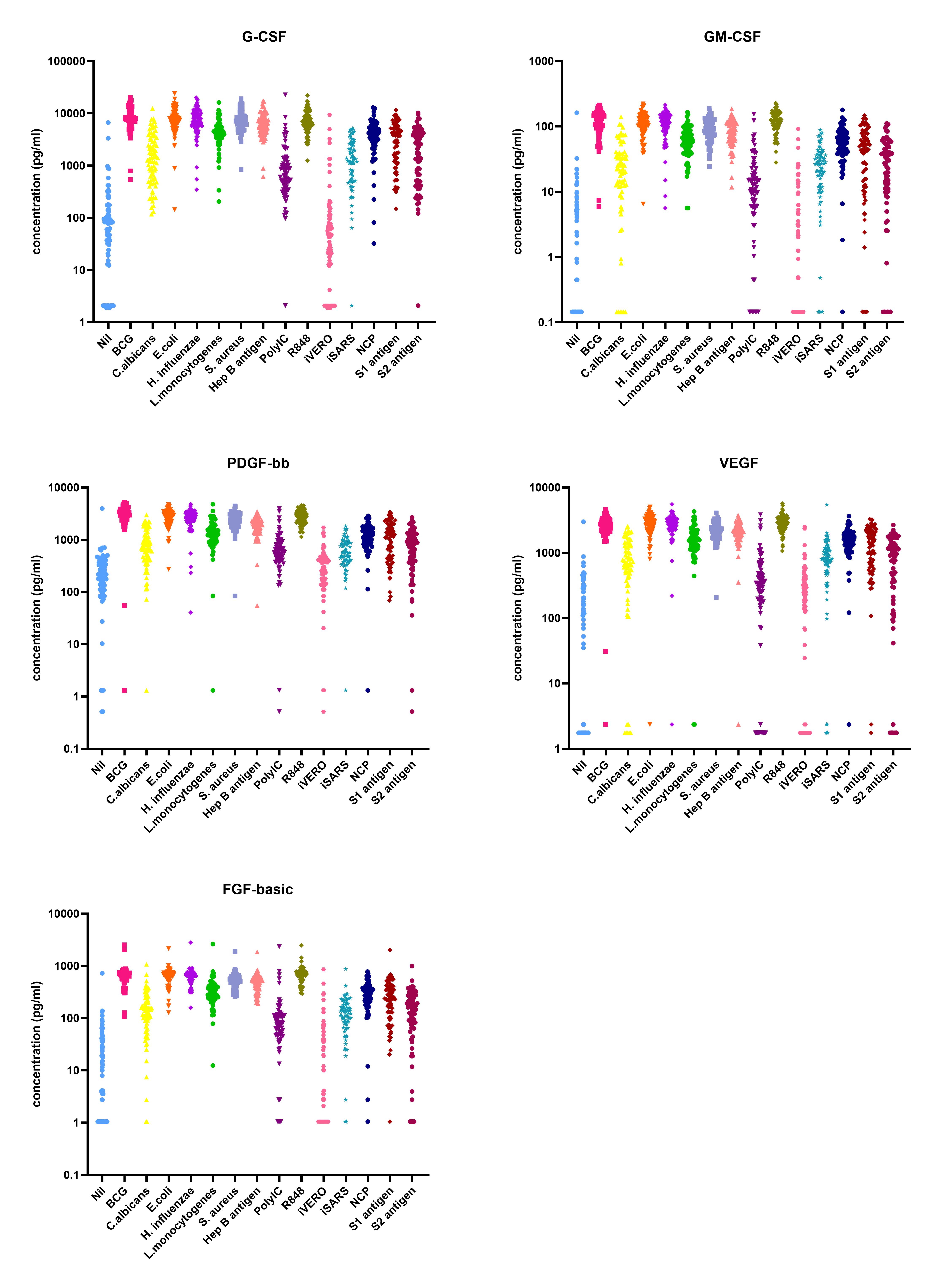

Supplement: Supplementary Figure 1 — Study timeline. Participants were requested to provide blood samples at two core visits and one optional visit. The first blood sample was taken just before, and on the same day, as the first BNT162b2 vaccination (V1), the second blood sample was taken 28 days after the second BNT162b2 vaccination (V2 + 28) and the optional third blood sample was taken 6 months after the second BNT162b2 vaccination (V2 + 182). The needle represents vaccination with BNT162b2, and the drops represent blood sampling. Time is indicated in days. [file DataSheet_1.zip › Supplementary Figure 2(B).jpg]

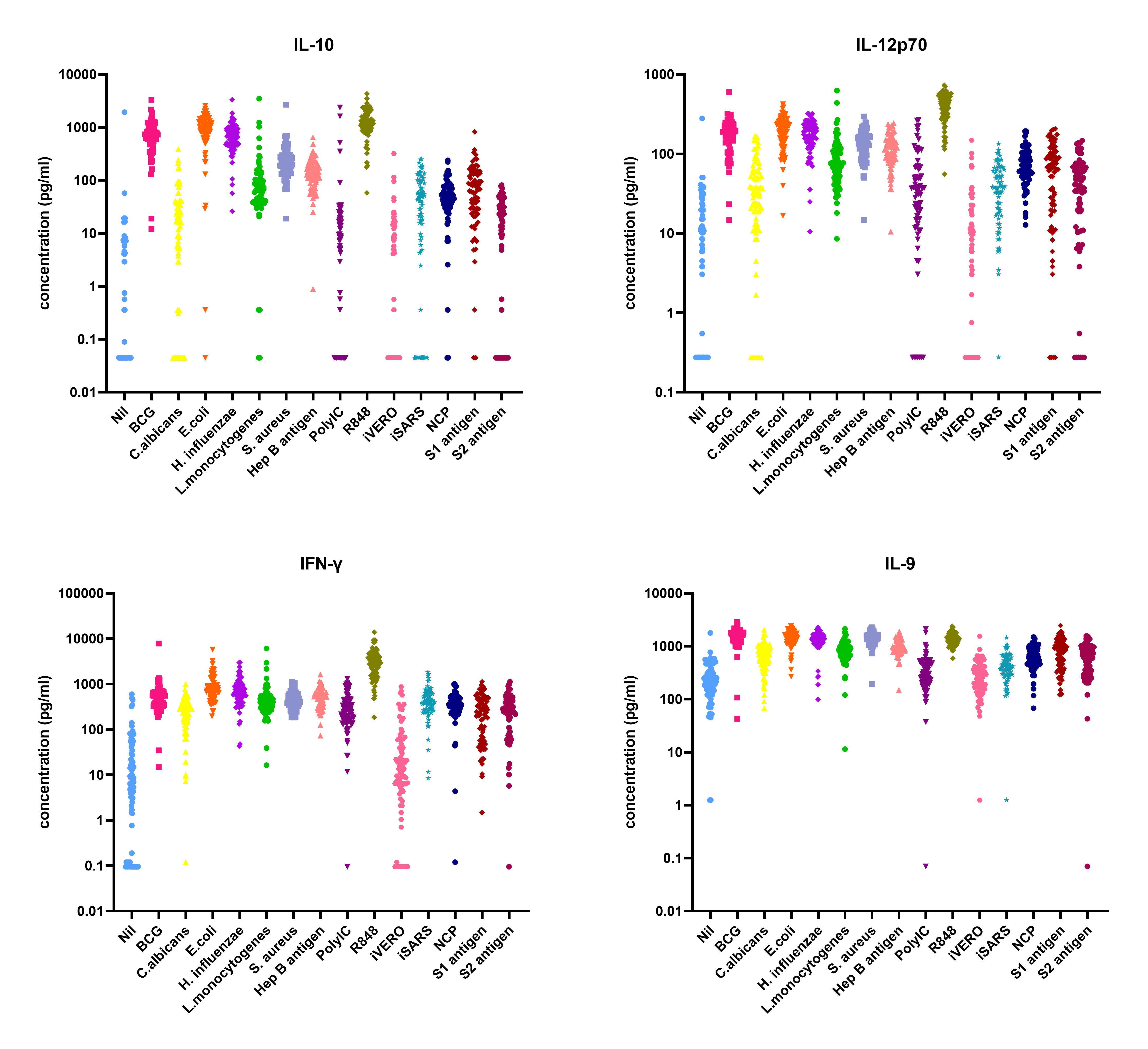

Supplement: Supplementary Figure 1 — Study timeline. Participants were requested to provide blood samples at two core visits and one optional visit. The first blood sample was taken just before, and on the same day, as the first BNT162b2 vaccination (V1), the second blood sample was taken 28 days after the second BNT162b2 vaccination (V2 + 28) and the optional third blood sample was taken 6 months after the second BNT162b2 vaccination (V2 + 182). The needle represents vaccination with BNT162b2, and the drops represent blood sampling. Time is indicated in days. [file DataSheet_1.zip › Supplementary Figure 2(C).jpg]

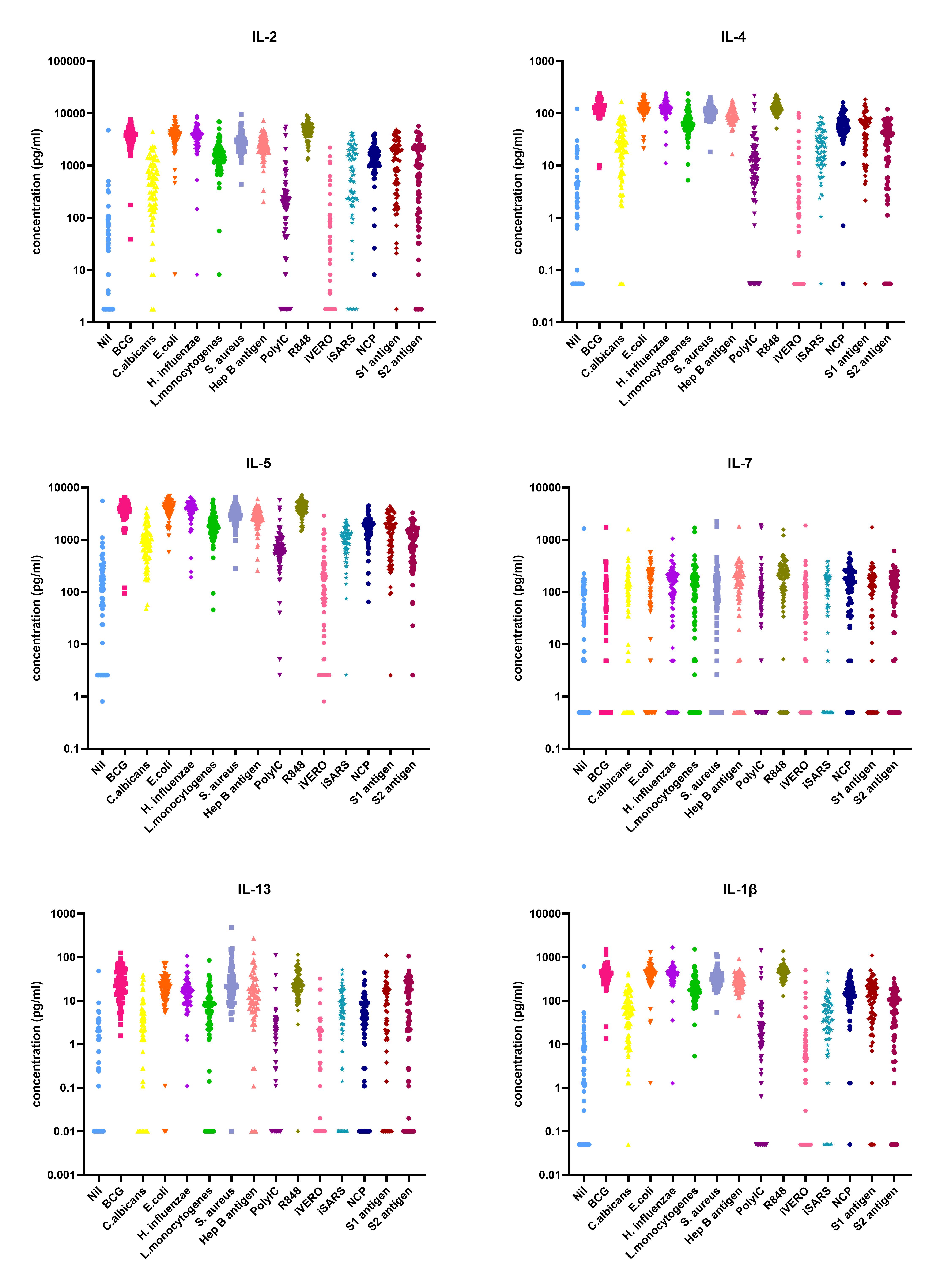

Supplement: Supplementary Figure 1 — Study timeline. Participants were requested to provide blood samples at two core visits and one optional visit. The first blood sample was taken just before, and on the same day, as the first BNT162b2 vaccination (V1), the second blood sample was taken 28 days after the second BNT162b2 vaccination (V2 + 28) and the optional third blood sample was taken 6 months after the second BNT162b2 vaccination (V2 + 182). The needle represents vaccination with BNT162b2, and the drops represent blood sampling. Time is indicated in days. [file DataSheet_1.zip › Supplementary Figure 2(D).jpg]

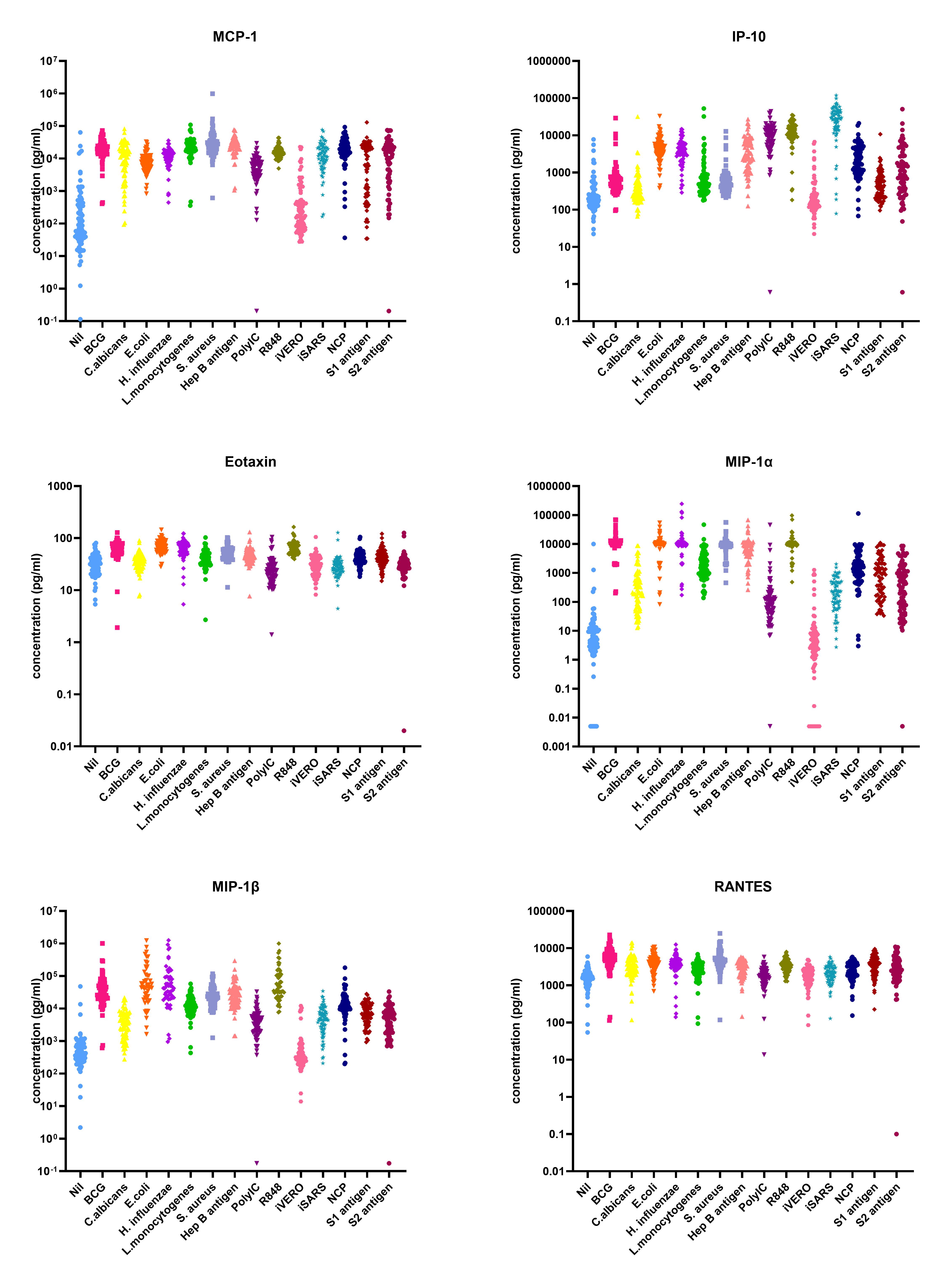

Supplement: Supplementary Figure 1 — Study timeline. Participants were requested to provide blood samples at two core visits and one optional visit. The first blood sample was taken just before, and on the same day, as the first BNT162b2 vaccination (V1), the second blood sample was taken 28 days after the second BNT162b2 vaccination (V2 + 28) and the optional third blood sample was taken 6 months after the second BNT162b2 vaccination (V2 + 182). The needle represents vaccination with BNT162b2, and the drops represent blood sampling. Time is indicated in days. [file DataSheet_1.zip › Supplementary Figure 2(E).jpg]

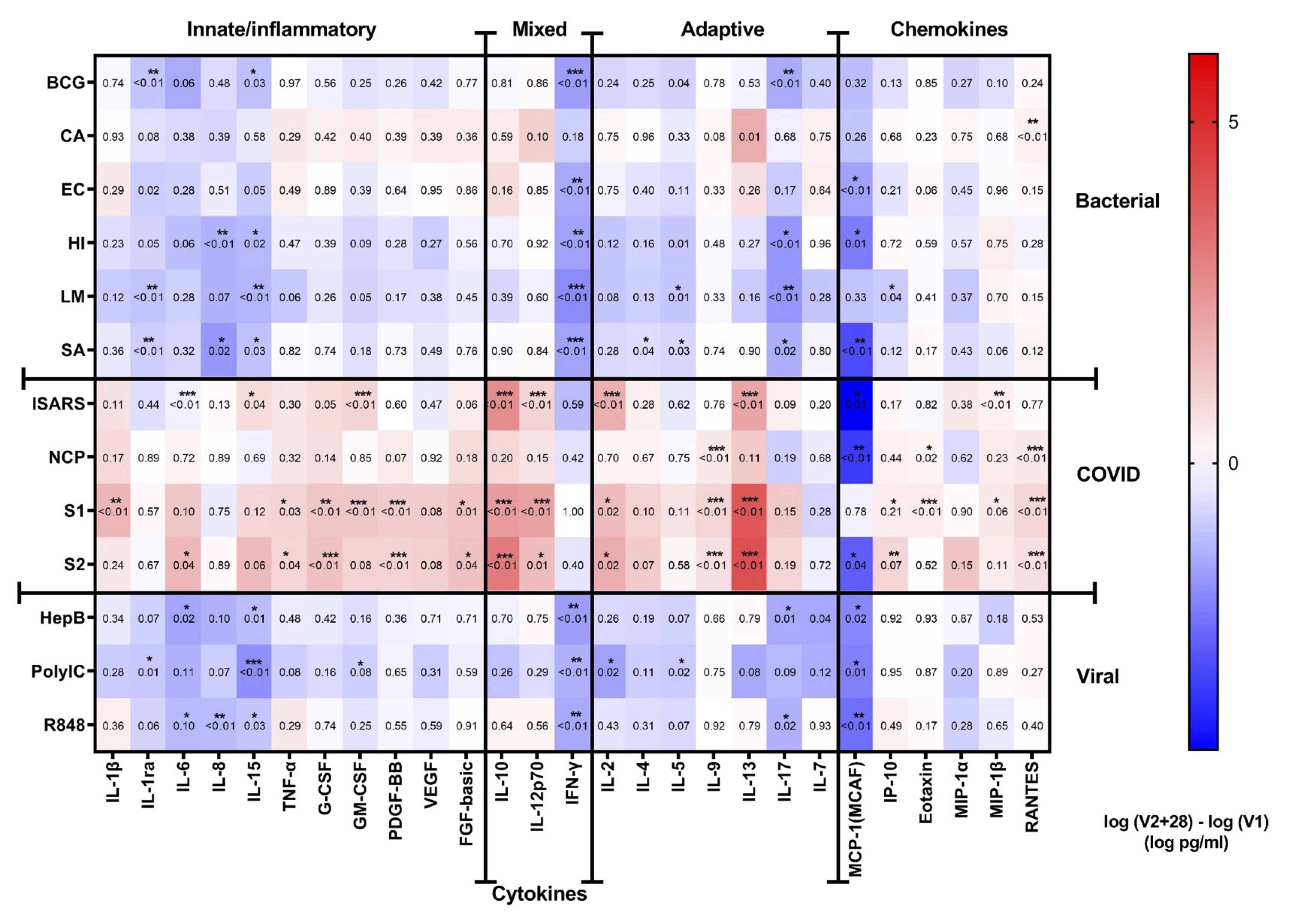

Supplement: Supplementary Figure 1 — Study timeline. Participants were requested to provide blood samples at two core visits and one optional visit. The first blood sample was taken just before, and on the same day, as the first BNT162b2 vaccination (V1), the second blood sample was taken 28 days after the second BNT162b2 vaccination (V2 + 28) and the optional third blood sample was taken 6 months after the second BNT162b2 vaccination (V2 + 182). The needle represents vaccination with BNT162b2, and the drops represent blood sampling. Time is indicated in days. [file DataSheet_1.zip › Supplementary Figure 3.jpg]
